# Complete Genome Sequences of Two Plant-Associated *Pseudomonas putida* Isolates with Increased Heavy-Metal Tolerance

**DOI:** 10.1128/genomeA.01330-17

**Published:** 2017-11-22

**Authors:** Joseph Nesme, Barbara Cania, Urška Zadel, Anne Schöler, Grażyna A. Płaza, Michael Schloter

**Affiliations:** a Research Unit for Comparative Microbiome Analysis, Department of Environmental Sciences, Helmholtz Zentrum München, Neuherberg, Germany; b Department of Environmental Microbiology, Institute for Ecology of Industrial Areas, Katowice, Poland

## Abstract

We report here the complete genome sequences of two *Pseudomonas putida* isolates recovered from surface-sterilized roots of *Sida hermaphrodita*. The two isolates were characterized by an increased tolerance to zinc, cadmium, and lead. Furthermore, the strains showed typical plant growth-promoting properties, such as the production of indole acetic acid, cellulolytic enzymes, and siderophores.

## GENOME ANNOUNCEMENT

The *Pseudomonas putida* complex is a broad group of *Gammaproteobacteria* including mostly isolates from environmental samples (1) and comprising several genomic species (2). *P. putida* isolates are characterized by a versatile metabolism allowing them to survive in a variety of different ecological settings. *P. putida* is also often found to be associated with plants and can actively enhance their growth and tolerance against abiotic and biotic stressors (3, 4), notably, elevated heavy-metal concentrations in soil (5–7).

*Pseudomonas putida* strains, namely, E41 and E46, were isolated from surface-sterilized roots of *Sida hermaphrodita* grown on the Institute for Ecology of Industrial Areas’ experimental field in Bytom, Upper Silesia, Poland (50°20′43.0″N, 18°57′19.6″E). Both strains were able to grow on tryptic soy agar (TSA) plates in the presence of either 8 mM zinc, 4 mM lead, or 2 mM cadmium, or with a combination of 4 mM zinc and 1 mM cadmium. They also express simultaneously four traits linked to plant growth-promoting rhizobia (PGPR) (i.e., indole acetic acid [IAA] synthesis, siderophores, HCN production, and phosphate solubilization). In the frame of this project, we were able to reconstruct the genomes of the two isolates consisting of a single circular chromosome replicon using PacBio RSII and PacBio Sequel technologies, which were further polished with Illumina MiSeq reads.

Genomic DNA from each strain was isolated using a commercially available kit (Macherey-Nagel GmbH, Düren, Germany). Strain E41 was sequenced using PacBio RSII technology by GATC Biotech (Konstanz, Germany) at 200-fold coverage. Sequence assembly was performed using the single molecule real-time (SMRT) portal interface and RS_HGAP_Assembly.2 protocol (Pacific Biosciences, Menlo Park, CA, USA) and produced a single contig. Strain E46 was sequenced using PacBio Sequel technology at 450-fold coverage at the Research Unit for Comparative Microbiome Analysis (Helmholtz Zentrum München, Neuherberg, Germany). Sequence assembly was performed using SMRT Link interface and Assembly (HGAP 4) analysis application (Pacific Biosciences) and produced a single contig. Genomic DNA from both strains was additionally sequenced using Illumina MiSeq. For MiSeq sequencing, libraries were prepared using the TruSeq DNA PCR-free library preparation kit (Illumina, San Diego, CA, USA), with genomic DNA fragmented using the Covaris E220 system, according to the manufacturer’s protocol, for a 1-kbp average insert size, and sequenced using MiSeq reagent kit version 3 (Illumina). The assembled reads of both strains were improved for small insertion-deletion and base correction using Pilon software version 1.22 (8) and BWA-MEM mapping of the corresponding Illumina MiSeq paired-end read data set with the parameters “-I 1000, -x intractg, -t 20.” The corrected assembly was then circularized using Circlator version 1.5.1. The Pilon-polished assembly and corrected PacBio reads obtained from HGAP intermediate output were used as input for Circlator, using default parameters “circlator all <assembly.fasta> <pacbio corrected reads.fasta> <output directory>” (9). The obtained genome sequence has been verified for circularity using overlapping contig ends. The linear sequence representation has been corrected so that the first open reading frame corresponds to the *dnaA* gene located next to the origin of replication. The genomes were submitted to the NCBI Prokaryotic Genome Annotation Pipeline (10) on October 2017.

### Accession number(s).

This whole-genome shotgun project has been deposited at DDBJ/EMBL/GenBank under the accession no. CP024085 and CP024086 for *Pseudomonas putida* strains E41 and E46, respectively.
